# Feasibility and Acceptability of a US National Telemedicine Curriculum for Medical Students and Residents: Multi-institutional Cross-sectional Study

**DOI:** 10.2196/43190

**Published:** 2023-05-08

**Authors:** Rika Bajra, Winfred Frazier, Lisa Graves, Katherine Jacobson, Andres Rodriguez, Mary Theobald, Steven Lin

**Affiliations:** 1 Division of Primary Care and Population Health Department of Medicine Stanford University School of Medicine Palo Alto, CA United States; 2 St. Margaret Family Medicine Residency Program University of Pittsburgh Medical Center Pittsburgh, PA United States; 3 Department of Family and Community Medicine Western Michigan University Homer Stryker M.D. School of Medicine Kalamazoo, MI United States; 4 Department of Family and Community Medicine University of Maryland School of Medicine Baltimore, MD United States; 5 Division of Family and Community Medicine Department of Humanities, Health, and Society Florida International University Herbert Wertheim College of Medicine Miami, FL United States; 6 Society of Teachers of Family Medicine Leawood, KS United States

**Keywords:** curriculum, distance education, graduate medical education, telemedicine, undergraduate medical education

## Abstract

**Background:**

Telemedicine use increased as a response to health care delivery changes necessitated by the COVID-19 pandemic. However, lack of standardized curricular content creates gaps and inconsistencies in effectively integrating telemedicine training at both the undergraduate medical education and graduate medical education levels.

**Objective:**

This study evaluated the feasibility and acceptability of a web-based national telemedicine curriculum developed by the Society of Teachers of Family Medicine for medical students and family medicine (FM) residents. Based on the Association of American Medical Colleges telehealth competencies, the asynchronous curriculum featured 5 self-paced modules; covered topics include evidence-based telehealth uses, best practices in communication and remote physical examinations, technology requirements and documentation, access and equity in telehealth delivery, and the promise and potential perils of emerging technologies.

**Methods:**

A total of 17 medical schools and 17 FM residency programs implemented the curriculum between September 1 and December 31, 2021. Participating sites represented 25 states in all 4 US census regions with balanced urban, suburban, and rural settings. A total of 1203 learners, including 844 (70%) medical students and 359 (30%) FM residents, participated. Outcomes were measured through self-reported 5-point Likert scale responses.

**Results:**

A total of 92% (1101/1203) of learners completed the entire curriculum. Across the modules, 78% (SD 3%) of participants agreed or strongly agreed that they gained new knowledge, skills, or attitudes that will help them in their training or career; 87% (SD 4%) reported that the information presented was at the right level for them; 80% (SD 2%) reported that the structure of the modules was effective; and 78% (SD 3%) agreed or strongly agreed that they were satisfied. Overall experience using the national telemedicine curriculum did not differ significantly between medical students and FM residents on binary analysis. No consistent statistically significant relationships were found between participants’ responses and their institution’s geographic region, setting, or previous experience with a telemedicine curriculum.

**Conclusions:**

Both undergraduate medical education and graduate medical education learners, represented by diverse geographic regions and institutions, indicated that the curriculum was broadly acceptable and effective.

## Introduction

Telemedicine—the delivery of health care remotely using telecommunication technology [1]—emerged at the forefront of clinical care during the COVID-19 pandemic. Over the last 20 years, known benefits include increased patient access (especially in underserved and rural areas), decreased health care costs, and high patient and physician satisfaction [2,3]. Although the pandemic unexpectedly accelerated the adoption of telemedicine [4], many academic medical centers are now purposefully developing strategies for the long-term integration of telemedicine and digital health tools into clinical care and medical education [5,6]. Furthermore, with the emergence of technologies such as remote patient monitoring, there is an urgency to train future physicians in the meaningful use of telemedicine in the context of a rapidly evolving health care landscape.

Recognizing a need for telemedicine education, the Association of American Medical Colleges (AAMC), the Liaison Committee on Medical Education, and the American Academy of Family Physicians (AAFP) recommended adoption of telemedicine into medical school and residency training before the pandemic [7]. Between 2018 and 2021, the number of US medical schools offering telemedicine education in a required or elective course dramatically increased from 58% to 90% [8]. Similarly, telemedicine use in residencies rapidly expanded once the Centers for Medicare and Medicaid Services extended reimbursement for telemedicine outside of rural areas and allowed remote precepting [9]. Proposed changes to the Accreditation Council for Graduate Medical Education family medicine program requirements state, for the first time, that resident patient encounters should include telemedicine visits [10].

Despite the expansion of telemedicine education at medical schools and residency programs, there are still significant telemedicine curricular gaps [11,12]. For example, while medical students express a desire to learn telemedicine best practices in undergraduate training [11], a 2020 survey of 156 internal medicine postgraduate year 1 (PGY-1) residents demonstrated that 74% of them did not receive dedicated telemedicine training during medical school, and only 12% of them felt “at least moderately” prepared to conduct telemedicine visits at the start of residency [12]. A 2021 survey of 213 residents (PGY-1 to PGY-7) representing 51 different specialties showed 72% felt that specific training in telemedicine was important for their careers [13].

Medical schools frequently cite a lack of faculty experience in telemedicine as a significant barrier to developing telemedicine education [14]. An additional barrier is the lack of a recognized gold standard for telemedicine training [15-18]. In response to this, the Society of Teachers of Family Medicine (STFM) formed a task force to create a national telemedicine curriculum for medical students and family medicine (FM) residents [19], using an expanded version of AAMC’s cross-continuum telemedicine competencies [20]. This study describes the feasibility and acceptability of this national telemedicine curriculum, covering 20 telemedicine competencies over 5 web-based modules, across a diverse group of undergraduate medical education and graduate medical education (GME) settings.

## Methods

### Curriculum Development

The STFM Telemedicine Task Force convened in June 2020 to develop a national curriculum for medical schools and FM residencies, covering foundational topics and best practices in telemedicine. Task force members included multidisciplinary medical educators and telehealth experts from diverse organizations, including the AAFP, AAMC, the US Department of Veterans Affairs, academic medical centers, and large health delivery systems across the country [19].

Task force members developed the telemedicine curriculum between September 2020 and August 2021. Curriculum development used Kern’s 6-step framework [21], including a targeted needs assessment, learning objectives mapped to AAMC competencies, incorporation of effective web-based educational strategies, and implementation as a multi-institutional pilot for evaluation. The needs assessment was conducted through a comprehensive literature review of existing telemedicine curricula. Learning objectives were mapped to AAMC’s telehealth competencies [20], and additional competencies were added by consensus decision-making [22]. Developed with the use of evidence-based principles in multimedia instruction [23,24], the modules incorporated instructional videos, animations, and interactive exercises to foster effective learning; modular content was organized into visually engaging screens for easy, self-paced scrolling on a laptop or mobile device. The modules prompted learners to apply, analyze, and synthesize learning concepts (hierarchical elements of Bloom’s taxonomy [25]) through interactive click-and-point exercises, reflective questions, and case-based medical decision-making.

**Figure 1:**
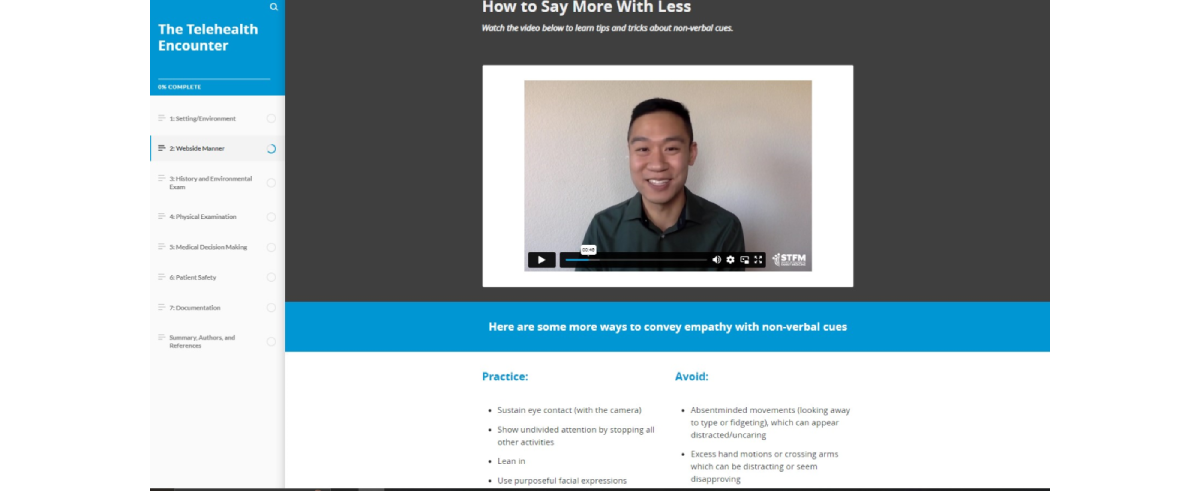
Screenshot of “The Telehealth Encounter” in Module 2.

**Figure 2:**
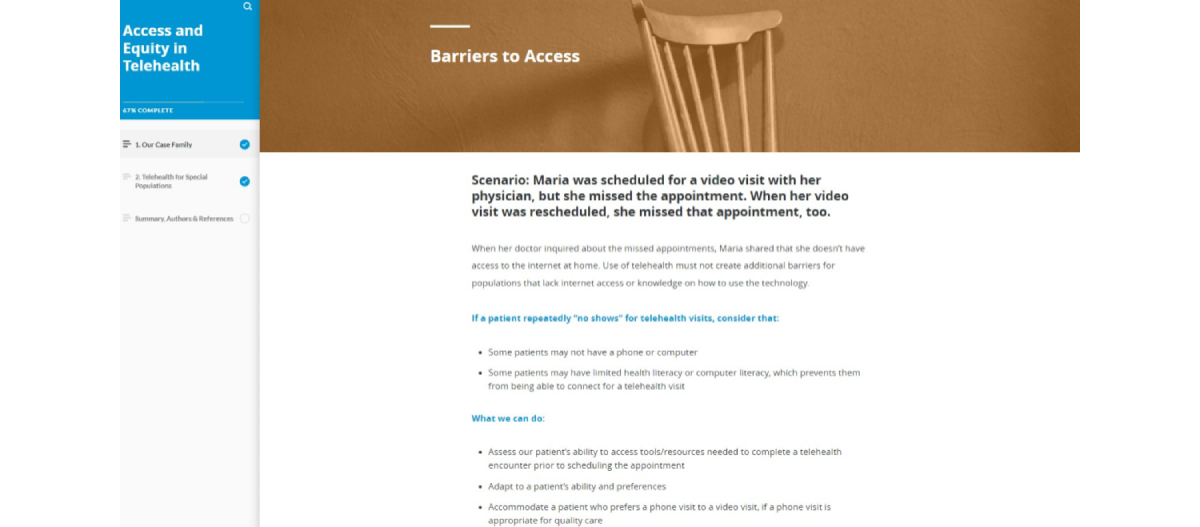
Screenshot of “Access and Equity in Telehealth” in Module 4.

Table 1 details the content of the 5-module curriculum. Module 1 (Intro to Telehealth) provides evidence-based telehealth uses. Module 2 (The Telehealth Encounter) reviews best practices in setting up a confidential, therapeutic environment, as well as “webside” manner, remote physical examinations, and medical decision-making. Module 3 (Requirements of Telehealth) covers technology requirements and documentation. Module 4 (Access and Equity in Telehealth) focuses on access and equity to mitigate bias, promote cultural competence, and address potential technology barriers. Module 5 (Future of Telehealth) addresses the promise and potential perils of emerging technologies. Figures 1 and 2 are representative screenshots of the modules; a short overview video of the curriculum can be found in the Multimedia Appendix 1.

**Table 1:** Overview of the Society of Teachers of Family Medicine national telemedicine curriculum, 2021: five comprehensive modules.

Module	AAMC^a^ competency domain	ACGME^b^ core competency and subcompetencies	Learning objectives	Teaching method in module
Introduction to telehealth	Patient safety and appropriate uses	Practice-based learning and improvement: investigate and evaluate patient care practices, appraise and assimilate scientific evidence Systems-based practice: coordinate patient care within the health system, incorporate considerations of cost awareness and risk/benefit analysis	Describe the appropriate uses of telehealth Discuss the benefits and limitations of telehealth Identify factors that impact patient and practice barriers to incorporating telehealth Explain the roles and responsibilities of team members in telehealth encounters	Evidence-based research on current telemedicine uses, risk and benefits Review of telemedicine barriers including patient readiness and access to technology Interactive point-and-click graphics and multiple-choice question
The telehealth encounter	Communication; data collection, and assessment	Interpersonal and communication skills: create and sustain a therapeutic relationship with patients and families Patient care and procedural skills: gather essential and accurate information, counsel patients and family members, make informed diagnostic and therapeutic decisions Medical knowledge: demonstrate an investigative and analytical approach to clinical problem solving and knowledge acquisition, apply medical knowledge to clinical situations	Establish a therapeutic environment and develop effective rapport with patients Obtain a history and conduct an appropriate physical examination through telehealth Incorporate information from the patient’s surroundings into the clinical assessment Apply appropriate medical decision-making in the context of providing care at a distance, including escalating care when necessary Complete documentation for telehealth encounters	Case-based teaching with standardized patient videos: learners assess therapeutic environment, clinical symptoms, and respond to multiple-choice and free response questions Interactive exercisesto navigate communication challenges (including sample scripts) and identification of health risks in environmental Tutorial videos on best practices for webside manner, physical examination, medical decision-making
Requirements for telehealth	Technology for telehealth; ethical practices and legal requirements (privacy regulations, informed consent, professional requirements)	Systems-based practice: advocate for quality patient care and optimal patient care systems, work in interprofessional teams to enhance patient safety and improve patient care quality	Describe the technology requirements for a telehealth encounter Resolve common telehealth technical issues List the documentation requirements Identify the key elements of an effective telehealth work environment	Point-and-click interactive exercises for technology troubleshooting Review of Health Insurance Portability and Accountability Act (HIPAA) compliance, documentation requirements including sample language and resources
Access and equity in telehealth	Access and equity (mitigate bias, promote health equity, address potential barriers to use)	Professionalism: demonstrate professional conduct and accountability, humanism, and cultural proficiency Interpersonal and communication skills: create and sustain a therapeutic relationship with patients and families	Describe how telehealth may mitigate or amplify socioeconomic gaps in health care access Assess and accommodate patients’ needs, preferences, and potential cultural, social, physical, cognitive, and linguistic/communication barriers to technology use Use telehealth to effectively deliver care for special populations (child/adolescent, geriatric patients with dementia or in a nursing home, patients at risk for intimate partner violence, LGBTQI patients, incarcerated patients, mental health care)	Interactive, case-based scenarios for telemedicine visits with pediatric and adolescent patients; dementia and nursing home patients; lesbian, gay, bisexual, transgender, queer, and intersex (LGBTQI); mental health patients; visits with interpreters Reflective questions on cultural competence, barriers to care, maintaining confidentiality
Future of telehealth	Technology for telehealth (Emerging technologies)	Practice-based learning and improvement: investigate and evaluate patient care practices, appraise and assimilate scientific evidence Systems-based practice: incorporate considerations of risk/benefit analysis, advocate for quality patient care and optimal patient care systems, participate in identifying system errors	Describe the current trends in telemedicine delivery models and new technologies Describe the types of technological innovations that may impact telemedicine in the future, including artificial intelligence Discuss methods of data acquisition Describe methods of interpreting healthcare data and subsequent utilization of this data	Review of emerging innovations (remote patient monitoring and artificial intelligence), for chronic care management and population health Evaluation of emerging technology with consideration to impact on physician-patient relationship, safety/quality, and ethical, equitable care

^a^AAMC is the Academic Association of Medical Colleges [26].

^b^ACGME is the Accreditation Council for Graduate Medicine Education [27].

### Multi-institutional Evaluation of Curriculum

A total of 17 medical schools and 17 FM residencies implemented the STFM national telemedicine curriculum between September 1 and December 31, 2021. Selected from 75 applicants that responded to an open call, applications were reviewed with attention to diverse characteristics including geography, private or public institution, practice setting, and previous exposure to telemedicine education. Selected institutions represented all 4 US census regions and 8 of 9 divisions (except for East South Central due to a lack of applicants from that area). The 25 represented states comprised 5 Western states, 8 Midwestern states, 8 Southern states, and 4 Northeastern states; 8 of the sites were in rural locations, 10 sites were urban, and 12 were suburban.

Upon completion of site selection, task force members conducted an informational meeting for site leads through Zoom to ensure an understanding of the study requirements. As part of the application process, each institution completed a prepilot survey and designated a site lead. The site leads completed the following tasks: (1) collated learner names to track curriculum completion, (2) implemented the modules as a required activity, (3) initiated follow-up with learners with incomplete work, and (4) submitted a postpilot survey on curricular implementation. Each institution distributed study information to learners, describing the use of deidentified and aggregated survey responses. A poststudy meeting was held in January 2022 with site leads to debrief on their experiences.

Learners completed a survey immediately after completing each of the 5 web-based modules, assessing their reaction and changes in knowledge, skills, and attitudes. Faculty site leads completed postpilot surveys that assessed faculty perception of the curriculum, including quality of each module, usefulness in developing telehealth skills, and overall satisfaction. All surveys used can be found in the Multimedia Appendices 2-4.

### Data Analyses

Statistical analysis was conducted using R statistical software (version 4.1.2; The R Foundation). Ordinal logistic regression analyses were performed for learner responses to 4 questions. Response variables ranged from either “strongly disagree” to “strongly agree” or from “way too basic” to “way too advanced.” Explanatory variables included institutions’ US census region, setting, and prior exposure to telemedicine curriculum. Chi-square tests were used to determine whether learners’ training level (eg, medical student or resident) was related to selecting “strongly agree” for gaining new knowledge, skills, or attitudes, the effectiveness of module structure, and overall satisfaction. We also tested whether learners’ training level was related to selecting “way too basic,” and, separately, “way too advanced” for appropriateness for the level of medical training. Results are presented for tests run with and without Yates’ correction.

### Ethics Approval

The AAFP Institutional Review Board approved this study (protocol #21-420, approved August 5, 2021).

## Results

### Results of Overall Curriculum

A total of 1203 learners, including 844 (70%) medical students and 359 (30%) FM residents, participated in the study (Table 2). Learners in all years were represented; third-year medical students represented the largest learner group, accounting for 36% (433/1203) of participants. 92% (1101/1203) of learners completed the entire curriculum (ie, all 5 modules). Most participants completed each module in 15-30 minutes (62%, SD 8%).

**Figure 3:**
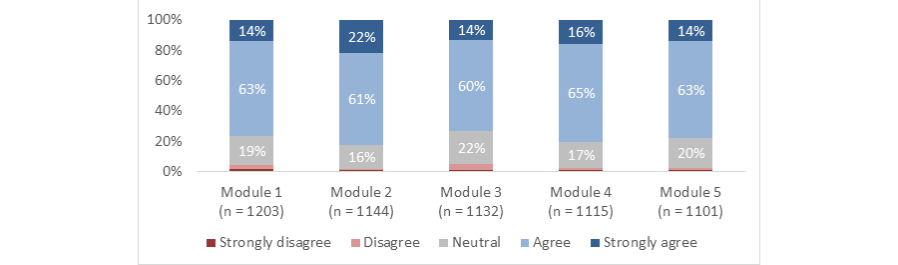
Responses to the statement, "By completing this module, I gained new knowledge, skills, and attitudes that will help me in my training or career.".

**Figure 4:**
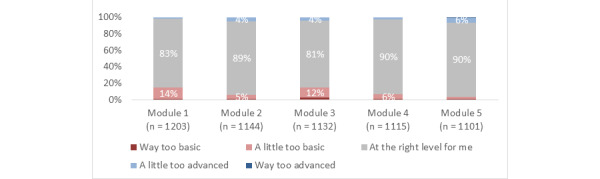
Responses to the statement, "Overall, for my level of medical training, the information in this module was.".

**Figure 5:**
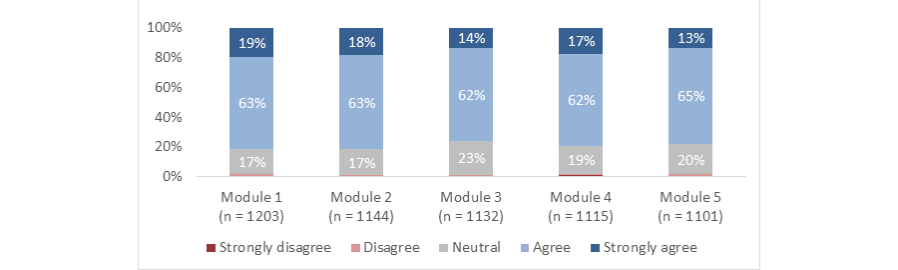
Responses to the statement, "Overall, the structure (layout, organization, etc) of this module was effective.".

**Figure 6:**
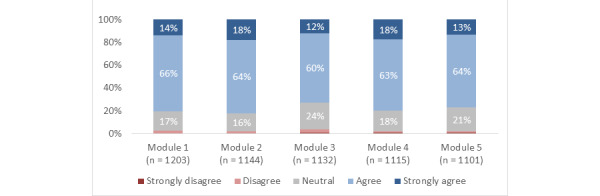
Responses to the statement, "Overall, I was satisfied with this module.".

Across the modules overall, 78% (SD 3%) of participants agreed or strongly agreed that they gained new knowledge, skills, or attitudes that will help them in their training or career; 87% (SD 4%) reported that the information presented was at the right level for them; 80% (SD 2%) reported that the structure (eg, layout and organization) of the modules was effective; and 78% (SD 3%) agreed or strongly agreed that they were satisfied (Figures 3-6).

**Table 2:** Demographics of participants in the national telemedicine curriculum evaluation, 2021.

			Medical students (N=844), n (%)		Family medicine residents (N=359), n (%)
**Level of training**					
	Year 1	224 (27)		120 (33)	
	Year 2	160 (19)		117 (33)	
	Year 3	433 (51)		108 (30)	
	Year 4	27 (3)		—^a^	
	Other	—		14 (4)	
**Region**					
	Midwest	321 (38)		96 (27)	
	Northeast	28 (3)		95 (26)	
	South	236 (28)		103 (29)	
	West	259 (31)		65 (18)	
**Setting**					
	Rural	196 (23)		87 (24)	
	Suburban	188 (22)		142 (40)	
	Urban	460 (55)		130 (36)	
**Previous exposure to telemedicine curriculum**					
	Yes	614 (73)		188 (52)	
	No	230 (27)		171 (48)	

^a^Not available.

### Results for Individual Modules

Using the completion of module 1 as baseline (1203/1203), the completion rate for modules 2 to 5, respectively, was 95% (1144/1203), 94% (1132/1203), 93% (1115/1203), and 92% (1101/1203). For modules 1 to 5, respectively, the proportion of participants who agreed or strongly agreed that they gained new knowledge, skills, or attitudes that will help them in their training or career was 77% (921/1203), 82% (942/1144), 73% (832/1132), 81% (898/1115), and 78% (855/1101); the proportion who reported that the information presented was at the right level for them was 83% (1003/1203), 89% (1,023/1144), 81% (913/1132), 90% (1009/1115), and 90% (994/1101); the proportion who reported that the structure of the module was effective was 82% (983/1203), 82% (935/1144), 76% (858/1132), 80% (888/1115), and 78% (862/1101); the proportion who agreed or strongly agreed that they were satisfied was 80% (965/1203), 82% (940/1144), 73% (822/1132), 80% (893/1115), and 77% (850/1101).

### Experience of Medical Students Versus FM Residents

The Overall experience did not differ significantly between medical students and FM residents on binary analysis (ie, agree or strongly agree vs disagree or strongly disagree). Medical students were significantly more likely than FM residents to strongly agree that they gained new knowledge, skills, or attitudes that will help them in their training or career for most of the modules (Modules 2 [*P*=.009], 3 [*P*=.003], 4 [*P*=.04], and 5 [*P*=.01]). FM residents were significantly more likely than medical students to report that the information presented was “way too basic” in modules 2 (*P*=.02), 4 (*P*=.007), and 5 (*P*=.007); but not 1 or 3. Medical students were significantly more likely than FM residents to strongly agree that the structure of the module was effective in modules 1 (*P*=.009), 2 (*P*=.02), 3 (*P*<.001), and 4 (*P*=.008); but not 5. Medical students were significantly more likely than FM residents to strongly agree that they were satisfied with modules 1 (*P*=.01), 2 (*P*=.01), 3 (*P*=.002), 4 (*P*<.001), and 5 (*P*<.001).

### Experience Versus Institutional Characteristics

No consistent statistically significant relationships were found between participants’ responses and their institution’s geographic region, setting (ie, urban, suburban, and rural), or previous experience with a telemedicine curriculum. When asked whether they were overall satisfied, as compared to those in rural settings, participants in urban settings were significantly less likely to agree or strongly agree for module 1 (OR 0.696, 95% CI 0.5-0.96; *P*=.03) and 2 (OR 0.718, 95% CI 0.52-0.99; *P*=.04), but not 3, 4, or 5. Compared to those at sites without a preexisting telemedicine curriculum, participants at sites with a telemedicine curriculum were significantly less likely to agree or strongly agree that they were satisfied for module 3 (OR 0.661, 95% CI 0.49-0.88; *P*=.005), but not 1, 2, 4, or 5.

### Faculty Evaluation

Faculty survey responses were received for 16 of 17 (94%) medical schools and 15 of 17 (88%) residencies. The faculty rated each module on a scale of 1-5 with 1 being poor and 5 being excellent. The overall mean rating for the entire curriculum was 4.2 (n=31); the range was 3.9-4.7. Both the medical school and residency faculty rated module 2 (The Telehealth Encounter) the highest at 4.7 and 4.4, respectively. The majority of faculty at medical schools (13/16, 81%) and residency programs (11/15, 73%) reported that the information presented was at the right level; 19% (3/16) of medical school faculty assessed the curriculum as a little too advanced, and 27% of residency faculty (4/15) assessed the curriculum as a little too basic. All medical school faculty were satisfied (3/16 very satisfied, 13/16 satisfied) with the curriculum and would recommend it to other medical schools (16/16, 100%). All residency faculty were satisfied (8/15 very satisfied, 5/15 satisfied, 2/15 somewhat satisfied) and the vast majority (14/15, 93%) would recommend it to other programs.

## Discussion

### Principal Findings

The STFM telemedicine curriculum was broadly accepted and well-received by learners at different stages of training and from multiple geographic regions and institutions, both with and without preexisting curricula. Most learners and faculty felt the curriculum was appropriate for their current needs—a surprising finding given the wide range of learners from early medical school to graduating residents—indicating that the curriculum can be tailored across the training continuum. For example, some faculty for preclerkship medical students implemented the curriculum in first- or second-year doctoring courses, highlighting history-taking and communication skills, while some clerkship-level faculty used the curriculum to develop clinical reasoning skills before clinical experiences or observed structured clinical examinations. Curricular implementation in GME includes use in intern orientation or group didactics, supplemented with case discussions to include more advanced applications, such as remote physical examination techniques and medical decision-making. In this manner, the curriculum functioned as a building block for a competency-based curriculum [28-30] in various ways, from acting as the entire telemedicine curriculum to being used as part of a flipped classroom, or grafted onto the existing curriculum to enhance content. The flexible, asynchronous nature, and feasible time frame for completion of the modules further optimized integration within crowded undergraduate medical education and GME training spaces.

### Comparison to Prior Work

While medical educators recognize the urgency to develop competency-based telemedicine curricula [28,29], the burden of creating curricula falls heavily on individual institutions, which may be particularly challenging for programs with limited resources. Prior studies indicate barriers to creating curricula include lack of faculty experience in this rapidly evolving field [14]. Furthermore, lack of standardized curricular content across institutions creates inconsistencies and gaps in telemedicine education [11,12]. Given potential limitations related to faculty resources, STFM’s “off-the-shelf” curricula offers a readily implementable tool for equitable access to telemedicine education with standardized, competency-based content.

### Strengths and Limitations

To our knowledge, our study is the largest multi-institutional evaluation of a telemedicine curriculum to date. Participating sites represented 25 states in all 4 US census regions with urban, suburban, and rural settings. In addition to geographic diversity, participants included both private and public institutions, as well as institutions with varying degrees of exposure to telemedicine education before our curriculum, from institutions with no previous exposure to those with preexisting curricula. In this diverse context, we found that both medical students and FM residents indicated that the STFM national telemedicine curriculum was effective and broadly acceptable.

We acknowledge several limitations to our study—first, our study primarily focused on evaluating learner experiences; however, some mitigation of potential bias has been made with faculty evaluations. Second, resident participants in our study were all from FM residencies. Although this curriculum does not address discipline-specific telemedicine applications, alignment with broader AAMC telemedicine competencies suggests that its value should extend well beyond the study group. Finally, while the asynchronous, self-paced format enabled flexibility to readily implement it across multiple institutions, we acknowledge that this learning format has limitations. Specifically, we were unable to assess higher level learning outcomes, such as whether learners changed behaviors, as this was not feasible at the scale of the study. Future research is needed to evaluate the curriculum’s impact on learner performance and outcomes. For example, this curriculum’s comprehensive learning objectives, mapped to AAMC’s telemedicine competencies, can serve as a springboard to develop standardized assessment checklists for observed structured clinical examinations or “live” clinical assessments.

### Future Directions

As medical educators innovate around telemedicine curricula, teaching future clinicians to consider ethical and societal implications of emerging technologies should not be overlooked. More than ever, learners require skills to critically assess new technologies, such as remote patient monitoring—with thoughtful consideration of their benefits and potential pitfalls. Given a widening “digital divide” between populations with and without access to these technologies [31,32], cultivating awareness and promoting equitable access cannot be overstated. The current STFM curriculum devotes 2 modules to inclusion of vulnerable populations and evaluating emerging technologies; future iterations of telemedicine curricula should continue to explore telemedicine’s role in mitigating—rather than exacerbating—existing health disparities, as more research in this area emerges.

The STFM national telemedicine curriculum was designed for medical students and residents. Inviting interprofessional colleagues to participate in the development and use of future iterations could facilitate interprofessional care. Telemedicine affords the opportunity for learners from various disciplines to participate in clinical care and enables the participation of learners who might otherwise be excluded from in-person learning. In this manner, when optimally and thoughtfully leveraged, telemedicine training can serve as a multifaceted opportunity for teachers and learners to explore equitable and learner- and patient-centered health systems that purposefully integrate telemedicine and digital health tools into clinical care and medical education.

### Conclusions

The STFM telemedicine curriculum was broadly accepted and well-received by learners at different stages of training and from multiple geographic regions and institutions in this large national study. It has the potential to serve as a foundation for a competency-based telemedicine curriculum for medical learners. Further research is warranted to evaluate the curriculum’s impact on learner performance and outcomes.

### Prior Presentations

Parts of this study were presented at the Society of Teachers of Family Medicine Conference on Medical Student Education (January 27-30, 2022) and the Society of Teachers of Family Medicine Annual Spring Conference (April 30-May 4, 2022).

## Appendix

Multimedia Appendix 1 A short overview video of the curriculum.

Multimedia Appendix 2 Learner Participant Survey.

Multimedia Appendix 3 Medical School Faculty Survey.

Multimedia Appendix 4 Residency Faculty Survey.

## Abbreviations

AAFP: American Academy of Family Physicians
AAMC: Association of American Medical Colleges
FM: family medicine
GME: graduate medical education
PGY: postgraduate year
STFM: Society of Teachers of Family Medicine

## Data Availability

The data sets generated and analyzed during the current study are available from the corresponding author on reasonable request.
