# Supplementary material for: Feasibility and Acceptability of a US National Telemedicine Curriculum for Medical Students and Residents: Multi-institutional Cross-sectional Study
Source: JMIR Med Educ. 2023 May 8;9:e43190. doi: 10.2196/43190 (PMC10203924; doi:10.2196/43190)
Supplement: Multimedia Appendix 2 [file mededu_v9i1e43190_app2.pdf]

Module 1: Intro to Telehealth

* 1. I am a:

- ◯ Year 1 medical student
- ◯ Year 2 medical student
- ◯ Year 3 medical student
- ◯ Year 4 medical student
- ◯ Other medical student
- ◯ Year 1 resident
- ◯ Year 2 resident
- ◯ Year 3 resident
- ◯ Other resident
- ◯ Faculty member
- ◯ Other (please specify)

[ ]

* 2. By completing this module I gained new knowledge, skills, and/or attitudes that will help me in my training and/or career.

◯ Strongly disagree ◯ Disagree ◯ Neutral ◯ Agree ◯ Strongly agree

* 3. Overall, for my level of medical training, the information in this module was:

◯ Way too basic ◯ A little too basic ◯ At the right level for me ◯ A little too advanced ◯ Way too advanced

Comments

[ ]

* 4. What was the most useful thing you learned from this module?

[ ]

* 5. Overall, the structure (layout, organization, etc.) of this module was effective.

◯ Strongly disagree ◯ Disagree ◯ Neutral ◯ Agree ◯ Strongly agree

* 6. Overall, I was satisfied with this module.

◯ Strongly disagree ◯ Disagree ◯ Neutral ◯ Agree ◯ Strongly agree

Comments (optional)

[ ]

* 7. How many minutes did it take you to complete this module? Enter a whole number. For example, if it took you an hour and 15 minutes, enter 75.

* 8. What could STFM do to improve this module?

* 9. What topics would you be interested in exploring further or receiving more training in? Select any/all that apply:

- [ ] Setting/Environment
- [ ] Physical Examination
- [ ] Webside Manner
- [ ] Medical Decision Making
- [ ] History and Environmental Exam
- [ ] Documentation
- [ ] Other (please specify)

Thank you for completing the module evaluation! Please enter your name below as a means of tracking individual completions of the survey. The information will not be used or published in any studies.

After entering your name, click next to download a certificate of completion for the module.

* 10. First Name

* 11. Last Name

Module 1: Intro to Telehealth

12. Click the following link to download your certificate of completion for:
Module 1: Intro to Telehealth

Thank you for completing this survey!
