# Supplementary material for: Feasibility and Acceptability of a US National Telemedicine Curriculum for Medical Students and Residents: Multi-institutional Cross-sectional Study
Source: JMIR Med Educ. 2023 May 8;9:e43190. doi: 10.2196/43190 (PMC10203924; doi:10.2196/43190)
Supplement: Multimedia Appendix 3 [file mededu_v9i1e43190_app3.pdf]

Telemedicine Curriculum Pilot Project Final Survey for Medical Schools

* 1. Institution Name:

* 2. Your name:

* 3. Your (primary) title:

* 4. Describe where in your curriculum the modules were implemented (e.g., required vs elective course; 1st year vs 3rd year; Sept. vs "do when you can" between Sept. and Dec.)

* 5. Describe - in detail - how the modules were integrated into your curriculum (e.g. done as a group, built into didactics, combined with other telemedicine education, independent learning)

* 6. Were your students allotted “protected time” to do the modules (e.g., time taken out of the course vs doing on their own time)?

- ◯ Yes
- ◯ No

* 7. Who at your medical school was assigned the STFM telemedicine modules? (check all that apply):

- ☐ Year 1 medical students
- ☐ Year 2 medical students
- ☐ Year 3 medical students
- ☐ Year 4 medical students
- ☐ Other (please specify)

* 8. How useful was this telemedicine curriculum in developing telehealth skills for medical students?

- ◯ Way too basic
- ◯ A little too basic
- ◯ At the right level
- ◯ A little too advanced
- ◯ Way too advanced

* 9. During the pilot project, did you require students to show you their certificates of completion to ensure they completed all the modules?

- ◯ Never
- ◯ Some of the time
- ◯ All of the time

* 10. How would you describe student engagement in these modules?

Not at all engaged — Extremely engaged

* 11. Based on your pilot experience, what advice might you offer to other medical schools regarding factors for successful implementation of these modules?

* 12. Rank the following modules in the order of value they added to your curriculum (with #1-ranked being the module that added the most value). If needed, go to https://stfm.org/telemedpilot to review the modules:

- ☐ Intro to Telehealth
- ☐ The Telehealth Encounter
- ☐ Requirements for Telehealth
- ☐ Access and Equity in Telehealth
- ☐ Future of Telehealth

* 13. On a scale of 1 to five, please rate the quality of the content of each of the modules. If needed, go to https://stfm.org/telemedpilot to review the modules:

	1 Poor	2	3	4	5 Excellent
Intro to Telehealth	○	○	○	○	○
The Telehealth Encounter	○	○	○	○	○
Requirements for Telehealth	○	○	○	○	○
Access and Equity in Telehealth	○	○	○	○	○
Future of Telehealth	○	○	○	○	○

* 14. Rate your overall satisfaction with the STFM telemedicine modules:

○ Dissatisfied

○ Not satisfied

○ Somewhat satisfied

○ Satisfied

○ Very satisfied

* 15. What could STFM do to improve the telemedicine modules?

* 16. What topics are missing from the modules? If you feel the topic list is complete/comprehensive as is, write "none."

* 17. Would you recommend STFM’s Telemedicine Curriculum to other medical schools?

○ Yes

○ No

Why or why not?
